# The Bromide of Ethyl as a Local Anæsthetic

**Published:** 1880-06

**Authors:** M. Terrillon, F. Peterson

**Affiliations:** The Societe De Chirurgie


### THE BROMIDE OF ETHYL AS A LOCAL ANÆSTHETIC.

BY M. TERRILLON, THE SOCIETE DE CHIRURGIE.

FROM THE FRENCH BY F. PETERSON, M. D.

I HAVE used this agent a dozen times for obtaining anæsthesia in the employment of the thermo-cautery. In all the cases I have seen, produced at the end of one or two minutes a blanching of the part, indicating anæsthesia of the skin. The pain was absolutely nothing. When it is necessary to prolong the anæsthesia, it may become sufficiently profound for opening abscesses situated one centimeter below the skin. The blanching of the skin is not an indispensable part of the anæsthesia, but so far this method has not deluded my hopes. I have, however, failed in one or two cases because certain atomizers gave much too fine a jet, and then the result was not obtained. I have had made an apparatus of a larger caliber which anæsthetizes the surface of the hand in one minute and some seconds. It is necessary that the beak of the atomizer be at a moderate distance from the hand, some eight or ten centimeters.—*La France Medicale.*

